# Relational and Organisational Factors Relating to Job Satisfaction Among Critical Care Nurses

**DOI:** 10.1111/nicc.70388

**Published:** 2026-02-07

**Authors:** Arum Lim, Seung Eun Lee

**Affiliations:** ^1^ Johns Hopkins School of Nursing Baltimore Maryland USA; ^2^ Yonsei University College of Nursing Seoul Republic of Korea

**Keywords:** critical care nursing, interprofessional relations, job satisfaction, leadership, safety management

## Abstract

**Background:**

The global nursing shortage poses significant challenges to healthcare systems. In high‐stakes environments such as intensive care units (ICUs), job satisfaction is a key determinant of nurse turnover.

**Aim:**

This study aimed to identify relational and organisational factors that influence job satisfaction among ICU nurses, as these factors remain underexplored in this context.

**Study Design:**

A cross‐sectional, correlational study used survey data collected from ICU nurses across 21 hospitals in Korea. Surveys on situation monitoring, collegial nurse–physician relationships and leader identification were collected as relational factors, and safety climate and workplace safety systems were investigated as organisational factors. Pearson correlations and multiple linear regression analyses were employed to investigate the relationship between the factors and job satisfaction.

**Results:**

This study included 200 ICU nurses. Pearson correlation analyses showed that all relational and organisational factors were significantly positively associated with job satisfaction. In multiple regression analyses adjusting for years of experience, leader identification showed the strongest positive association with job satisfaction (*β* = 0.328, *p* < 0.001), followed by safety climate (*β* = 0.160, *p* = 0.041), workplace safety systems (*β* = 0.153, *p* = 0.023) and collegial nurse–physician relationship (*β* = 0.139, *p* = 0.033). Situation monitoring was not significantly associated with job satisfaction (*β* = 0.044, *p* = 0.554).

**Conclusions:**

Job satisfaction among ICU nurses was significantly associated with both relational and organisational factors. In particular, strong identification with nurse leaders, positive collegial relationships with physicians, a favourable safety climate and the presence of systematic workplace safety procedures were all significant factors. These findings highlight the importance of nurses' identification with their leaders, interprofessional collaboration and a supportive safety environment in enhancing job satisfaction in high‐acuity care settings.

**Relevance to Clinical Practice:**

Healthcare organisations should promote interprofessional collaboration between nurses and physicians, as well as relationships with nurse leaders and prioritise cultivating a strong safety culture and system for patients and healthcare staff.

## Impact Statements

- What is known about this topic
  ○ Nurses' job satisfaction is a key determinant of turnover in high‐stakes settings such as intensive care units (ICUs).
  ○ Prior ICU research has focused on individual determinants, whereas relational and organisational influences remain underexplored.
- What this paper adds
  ○ Relational factors, particularly stronger identification with leaders and positive nurse–physician relationships, were positively associated with ICU nurses' job satisfaction.
  ○ ICU nurses' more positive perceptions of the unit's safety climate and safety systems were associated with higher job satisfaction.

## 1 Introduction

The global shortage of nurses presents significant challenges to healthcare systems, especially in intensive care units (ICUs), where patient outcomes depend heavily on highly skilled nursing care [1, 2, 3]. Critical care nurses manage life‐threatening conditions and complex clinical situations [4], high demands of their roles often result in elevated stress and emotional exhaustion [3]. In the United States, 60% of critical care nurses report experiencing inadequate staffing levels in their departments [3]. Globally, 28% of ICU nurses intend to leave their current positions [5], and high turnover rates among ICU nurses are contributing to the concerning staff shortage in the acute care nursing workforce [6]. This ongoing shortage further strains healthcare systems and diminishes both patient care quality [7] and organisational performance [8].

Job satisfaction has emerged as a key determinant of nurses' turnover in high‐stakes environments [3], such as critical care settings, where effective decision‐making and interpersonal dynamics are critical [2]. Job satisfaction is shaped by various factors, including individual, relational and organisational components [3, 9, 10]. Individual factors typically refer to personal or psychological attributes, such as demographic, career experience, perceived job stress and burnout [9, 10, 11]. Relational factors involve interpersonal dynamics, including relationships with colleagues and leaders [9, 10, 11]. Organisational factors include broader system‐level conditions, such as safety climate and the presence of formal safety procedures and systems [9, 10, 11, 12]. Although job satisfaction is influenced by individual, relational and organisational aspects, prior research has largely focused on individual determinants, leaving relational and organisational influences underexplored in the context of ICU nursing [9, 10, 11].

## 2 Background

Given the collaborative and interdisciplinary characteristics of critical care settings, relational factors, such as teamwork [9], quality of nurse–physician relationships [13] and relationships with their leaders [14], are particularly crucial in shaping ICU nurses' job satisfaction. Effective collaboration among nurses and between nurses and physicians enhances communication, facilitates decision‐making and improves overall patient care quality, whereas poor interprofessional relationships can increase stress and reduce job satisfaction [2, 3]. A recent publication emphasised that recognition of ICU nurses' roles and responsibilities, such as clinical decision‐making and continuous patient care, is essential for high‐quality teamwork and reducing turnover [2]. One of the core elements of teamwork among fellow nurses in high‐acuity settings in ICUs is situation monitoring, which plays a critical role in ensuring situational awareness and anticipating the needs of fellow team members [15]. As the boundaries for exercising nurses' clinical judgement should be clearly defined within each ICU team, a collegial relationship between nurses and physicians has also been highlighted [2]. Furthermore, how much nurses view their managers as respectful, trustworthy role models who align with their professional values, which refers to leader identification [16], is expected to play a pivotal role in influencing job satisfaction [3, 17]. A previous review reported that interpersonal factors, such as co‐worker interaction and professional relationships, were positively related to nurses' job satisfaction, but these findings were not specific to ICU nurses [11]. Similarly, a systematic review on ICU nurses' job satisfaction revealed a positive relationship between teamwork and job satisfaction, yet it did not explore specific elements of teamwork or the role of leader identification [9]. Consequently, the relationships of job satisfaction with collegial nurse–physician relationships, situation monitoring and leader identification remain underexplored in critical care settings.

In addition to relational factors, organisational factors—specifically safety climate and workplace safety systems—are vital in influencing nurses' job satisfaction [18, 19]. Safety climate refers to the shared perception among staff that safety is prioritised within the organisation, shaping their attitudes and behaviours towards safety practices [20]. Workplace safety systems involve the concrete policies, protocols and resources in place to manage and mitigate safety risks in the work environment [21]. These systems ensure that safety procedures are clearly defined, readily accessible and effective in protecting both staff and patients [20, 21]. Critical care nurses work in some of the most demanding healthcare environments, characterised by high patient acuity, fast‐paced decision‐making and frequent exposure to life‐and‐death situations [3, 22]. In such high‐stress environments, a strong safety climate helps build trust and reduces job‐related stress of ICU nurses [23]. Nurses' sense of security and trust in the well‐structured workplace safety systems strengthens their perception of clear procedural guidance and institutional support, alleviating uncertainty and reducing occupational injuries [24]. Together, these elements contribute to reduced occupational stress, which shapes nurses' job satisfaction [23]. Although both factors are recognised as important, their distinct yet complementary roles in shaping ICU nurses' job satisfaction have been relatively underexplored in prior research.

Building on the conceptual importance of these relational and organisational factors, this study sought to empirically examine their associations with ICU nurses' job satisfaction. Although previous research has primarily focused on individual‐level factors, such as demographics and psychological characteristics [9, 10, 11], few studies have integrated both relational and organisational factors in a single model. Furthermore, a systematic review of ICU nurses' job satisfaction highlighted the limited use of multivariate statistical approaches in this area [9].

## 3 Aims and Objective

This study aimed to examine the relationships between ICU nurses' job satisfaction and key relational and organisational factors, including nurse–physician relationships, situation monitoring, leader identification, safety climate and workplace safety systems, using multiple linear regression analysis. The objective of this study was to provide empirical insights that may contribute to a better understanding of nurses' job satisfaction in critical care settings by examining these relational and organisational factors.

## 4 Design and Methods

### 4.1 Setting and Sample

This cross‐sectional, correlational study is a secondary analysis of survey data originally collected between February and June 2022, as part of a larger web‐based survey of hospital nurses recruited from multiple regions across Korea [25]. This secondary analysis included all eligible participants from the original dataset. Eligible participants included ICU nurses with at least 6 months of clinical experience who were directly involved in patient care; those in managerial roles were excluded. As the sample size was determined for the original study, no additional a priori power analysis was conducted for this secondary analysis. Instead, a post hoc power analysis for multiple linear regression was performed, indicating an achieved statistical power exceeding 95%, assuming a medium effect size and a Type 1 error rate of 0.05.

### 4.2 Data Collection Tools and Measures

Job satisfaction was measured using the three‐item Job Satisfaction Subscale from the Michigan Organizational Assessment Questionnaire, originally developed by Cammann [26] and subsequently validated by Bowling and Hammond [27]. An example item is, ‘All in all, I am satisfied with my job’. Participants rated their responses on a 5‐point Likert scale (1 = *strongly disagree* to 5 = *strongly agree*), with higher scores reflecting greater job satisfaction. In this study, the Cronbach's alpha for the scale was 0.83.

Situation monitoring was assessed using the seven‐item Situation Monitoring Subscale from the Teamwork Perceptions Questionnaire [28]. A sample item is, ‘Staff effectively anticipate each other's needs’. Responses were scored on a 5‐point Likert scale (1 = *strongly disagree* to 5 = *strongly agree*), with higher scores indicating higher levels of situation monitoring. In this study, the Cronbach's alpha for the scale was 0.87.

Collegial nurse–physician relationship was measured using the three‐item Collegial Nurse–Physician Relations subscale of the Practice Environment Scale of the Nursing Work Index [29]. An example item is, ‘Physicians and nurses have good working relationships’. Responses were rated on a 4‐point Likert scale (1 = *strongly disagree* to 4 = *strongly agree*), with higher scores indicating more positive collegial relationships between nurses and physicians. In this study, the Cronbach's alpha for the scale was 0.89.

Leader identification was assessed using a validated 7‐item scale developed by Shamir et al. [16], designed to measure the extent to which individuals identify with their leader. A sample item is, ‘I trust his judgment and decisions completely’. Responses were collected on a 5‐point Likert scale (1 = *strongly disagree* to 5 = *strongly agree*), with higher scores indicating a stronger identification with the leader. In this study, the Cronbach's alpha for the scale was 0.96.

Safety climate was measured using the seven‐item Safety Climate subscale from the Safety Attitudes Questionnaire [20]. A sample item is, ‘I would feel safe being treated here as a patient’. Responses were scored on a 5‐point Likert scale (1 = *strongly disagree* to 5 = *strongly agree*), with higher scores reflecting a more positive perception of the safety climate within the work unit. In this study, the Cronbach's alpha for the scale was 0.87.

Workplace safety systems were assessed using the two‐item tool developed by Neal et al. [21]. A sample item is, ‘There are systematic procedures in place for preventing breakdowns in workplace safety’. Responses were measured on a 5‐point Likert scale (1 = *strongly disagree* to 5 = *strongly agree*), with higher scores indicating stronger perceptions of workplace safety systems. In this study, the Cronbach's alpha for the scale was 0.89.

Demographic and hospital‐related variables were collected. Demographic variables included gender, age, educational level, years of nursing experience, unit tenure and hospital tenure. Hospital‐related variables included location, teaching status, type and ownership.

### 4.3 Data Analysis

Descriptive statistics were used to summarise nurses' demographic and hospital characteristics, as well as key study variables. Pearson's bivariate correlations were calculated to examine the relationships among key study variables. Multicollinearity was assessed using variance inflation factor values, all of which were below 10, indicating no significant multicollinearity [30]. Multiple linear regression analysis was then performed to investigate the effects of independent variables on job satisfaction, adjusting for nurses' years of experience as a potential confounder given its known association with job satisfaction [11]. Data were analysed using STATA version 16.1, with statistical significance set at *p* < 0.05.

### 4.4 Ethics and Institutional Approvals

All participants were informed that their participation was voluntary and that they could withdraw from the study at any time without penalty. Informed consent was obtained electronically at the time of data collection. All data were collected anonymously and analysed in de‐identified form. The secondary data analysis was conducted in accordance with the principles of the Declaration of Helsinki and received ethical approval from the Yonsei University Health Systems Ethics Committee (no.: 4‐2024‐1406) on 31 December 2024.

## 5 Results

### 5.1 Sample and Hospital Characteristics

The analytic sample consisted of 200 nurses working in ICUs across 21 hospitals in Korea. In the original study, which recruited nurses from all clinical units, the response rate was 72.8% [25]. As shown in Table 1, most (88%) participants were female, with a mean age of 31.1 years (SD = 6.0). The majority of participants (94.5%, *n* = 193) held a baccalaureate or higher degree. The average nursing experience was 7.2 years (SD = 6.0), with a mean hospital tenure of 6.4 years (SD = 5.7) and unit tenure of 4.5 years (SD = 4.1).

**TABLE 1:** Sample and hospital characteristics (*N = 200*).

Characteristics	Mean ± SD or *N* (%)
Sample characteristics	
Age (years)	31.06 ± 6.02
Gender	
Female	176 (88.0)
Male	24 (12.0)
Education level	
College diploma	7 (3.5)
Bachelor's degree	173 (86.5)
Master's degree or higher	20 (10.0)
Nursing experience (years)	7.15 ± 6.02
Hospital tenure (years)	6.36 ± 5.71
Unit tenure (years)	4.45 ± 4.12
Hospital characteristics	
Location	
Seoul	37 (18.5)
Gyeonggi	74 (37.0)
Gyeongsang	34 (17.0)
Jeolla	20 (10.0)
Chungcheong	15 (7.5)
Gangwon	10 (5.0)
Jeju	10 (5.0)
Type of hospital	
Tertiary hospital	124 (62.0)
General hospital	76 (38.0)
Teaching status	
Teaching hospital	195 (97.5)
Non‐teaching hospital	5 (2.5)
Ownership	
Public	57 (28.5)
Private	143 (71.5)

Abbreviation: SD, standard deviation.

Participants were recruited from hospitals across various regions of Korea, as presented in Table 1. Of these, 55% were from the capital region, with the remainder from non‐capital regions. The regional distribution of the sample was generally aligned with the national distribution of ICU facilities in Korea, supporting the representativeness of the sample [31]. Additionally, 97.5% worked in teaching hospitals, and by ownership type, 71.5% were employed in private hospitals.

### 5.2 Bivariate Correlation Results

Table 2 provides the means, standard deviations and Pearson's bivariate correlations for the key study variables. Job satisfaction was significantly positively correlated with situation monitoring (*r* = 0.295, *p* < 0.001), collegial nurse–physician relationship (*r* = 0.360, *p* < 0.001), leader identification (*r* = 0.355, *p* < 0.001), safety climate (*r* = 0.397, *p* < 0.001) and workplace safety systems (*r* = 0.372, *p* < 0.001).

**TABLE 2:** Correlations, means and standard deviations for the key study variables.

Variables	Mean	SD	1	2	3	4	5	6
1. Job satisfaction	3.18	0.74	—					
2. Situation monitoring	3.75	0.51	0.295*	—				
3. Collegial nurse–physician relationship	2.59	0.64	0.360*	0.252*	—			
4. Leader identification	3.36	0.82	0.355*	0.403*	0.267*	—		
5. Safety climate	3.59	0.51	0.397*	0.639*	0.313*	0.473*	—	
6. Workplace safety systems	3.51	0.72	0.372*	0.368*	0.352*	0.314*	0.445*	—

*Note:* Correlation: Pearson correlation coefficient.

Abbreviation: SD, standard deviation.

* *p* < 0.001.

### 5.3 Multiple Regression Analysis Results

As shown in Table 3, leader identification had the strongest positive association with job satisfaction (*β* = 0.328, *p* < 0.001), followed by safety climate (*β* = 0.160, *p* = 0.041), workplace safety systems (*β* = 0.153, *p* = 0.023) and collegial nurse–physician relationship (*β* = 0.139, *p* = 0.033). Situation monitoring showed no significant relationship with job satisfaction (*β* = 0.044, *p* = 0.554).

**TABLE 3:** Multiple regression model for job satisfaction.

Variables	B	SE B	*β*	95% CI	*p*
Situation monitoring	0.057	0.097	0.044	−0.134, 0.249	0.554
Collegial nurse–physician relationship	0.163	0.008	0.139	0.013, 0.313	0.033
Leader identification	0.268	0.055	0.328	0.160, 0.376	< 0.001
Safety climate	0.224	0.109	0.160	0.009, 0.439	0.041
Workplace safety systems	0.134	0.058	0.153	0.018, 0.249	0.023

*Note:* The results were adjusted for nurses' years of experience.

Abbreviations: β, standardised coefficient; B, unstandardised coefficient; CI, confidence interval; SE, standard error of B.

## 6 Discussion

This study examined how relational and organisational factors are associated with job satisfaction among ICU nurses, who work in high‐stress and high‐complexity environments. After adjusting for nurses' years of clinical experience, leader identification, collegial nurse–physician relationships, safety climate and workplace safety systems emerged as factors that were significantly linked to job satisfaction. These results contribute to a more comprehensive understanding of how workplace dynamics influence ICU nurses' job satisfaction, expanding the focus beyond prior research that has largely emphasised individual characteristics.

Among the relational factors, leader identification showed the strongest positive association with job satisfaction. This finding suggests that ICU nurses who perceive their leaders as trustworthy, respectful and aligned with their own professional values are more likely to feel satisfied with their jobs. Although prior research in the United States and Brazil has highlighted the impact of leadership behaviours, such as transformational or authentic leadership, on nurse satisfaction [3, 32], few studies have focused on how leader identification itself contributes to job satisfaction in ICU contexts. Unlike leadership style, which reflects a leader's actions, leader identification centres on the nurse's psychological connection and sense of shared identity with their leader [16]. Such identification may foster trust, motivation and a sense of professional affirmation, all of which are important in high‐stress settings, such as ICUs. A study in the United Kingdom reported that role modelling with their leaders mediated the relationship between leader identification and higher job satisfaction and ultimately reduced turnover intent [33]. This finding underscores the importance of leadership development, which can equip nurse leaders with the knowledge, expertise and delegation skills required in complex ICU situations, while also strengthening effective role modelling and leader identification [34].

Moreover, consistent with evidence from previous review papers examining job satisfaction among nurses in acute hospital settings [9, 11], positive collegial nurse–physician relationships were associated with higher levels of job satisfaction among ICU nurses. Prior papers from countries in Europe and North America highlight the importance of effective interprofessional collaboration in creating a supportive work environment and enhancing nurse satisfaction [2, 3]. In critical care units, where managing complex patient needs demands seamless teamwork and interprofessional collaboration [35], respect between nurses and physicians reduces workplace conflict, improves communication and enhances the quality of care [13]. Moreover, shared care plans and interdependence between nurses and physicians can enhance responsibility for applying clinical judgment, another critical factor that positively contributes to job satisfaction [36]. Under many situations requiring immediate critical decisions, nurses can be more confident with real‐time decision‐making, which subsequently leads to work efficiency and safety [37]. Respected professional boundaries, which enable nurses to make decisions and hold themselves accountable, can also ensure that nurses communicate subtle clues they notice at the bedside regarding patient conditions, thereby fostering open discussions and professional interactions with other team members [37]. As shared goals, shared knowledge and mutual respect are important characteristics of effective relationships across diverse roles in health care, and these characteristics are not limited to the nurse–physician relationship [38], the concept of collegial relationships can be broadened to include relationships with other provider groups. These collaborative dynamics not only support the delivery of patient‐centred care but also contribute to a more positive work environment [3], which in turn could positively impact job satisfaction.

Safety climate also emerged as a significant organisational factor relating to ICU nurses' job satisfaction. When nurses perceive that their organisation actively prioritises safety, they may experience greater trust in their work environment and reduced job stress [18]. In high‐acuity settings, such as ICUs, where the potential consequences of clinical errors are severe [39], a strong safety climate can alleviate emotional strain and foster a sense of security [18]. This, in turn, may enhance job satisfaction by reinforcing the belief that their work environment supports safe and effective care delivery [23]. Moreover, safety climate that encourages employees to report errors and incidents, and to discuss and seek feedback [40], can also increase job satisfaction by promoting employees' positive work‐related motivation that makes them dedicated to their work [41]. Additionally, safety climate influences individuals' safety behaviours, such as speaking out about potential safety concerns and compliance with safety guidelines, which makes them feel more involved and contributes to patient safety, ultimately improving their job satisfaction [42].

Workplace safety systems were also significantly associated with job satisfaction among ICU nurses. These systems, which include structured protocols and preventive procedures, are designed to minimise occupational hazards and promote staff well‐being [21]. ICU nurses are frequently exposed to both psychological and physical risks, such as secondary traumatic experiences, problems, musculoskeletal injuries and hazardous substances during high‐risk procedures and routine tasks [43, 44, 45]. The presence of systematic safety procedures may enhance nurses' perceptions of institutional support and reduce anxiety about workplace hazards [46, 47]. Our findings suggest that when nurses perceive their organisation as actively safeguarding their safety through formal systems, they may experience greater confidence in the work environment and improved job satisfaction. These systems not only ensure a safe working environment but also reduce stress related to safety concerns [23], enabling nurses to perform their roles with greater focus and confidence. By prioritising staff safety through effective procedures, workplace safety systems foster a supportive organisational environment that can directly enhance job satisfaction.

Although previous reviews of studies conducted across multiple countries have demonstrated a positive association between teamwork and ICU nurses' job satisfaction [9, 11], situation monitoring, a key component of teamwork, was not significantly linked to job satisfaction in our multivariate analysis. Situation monitoring involves the continuous observation of clinical environments and team members to maintain awareness and anticipate needs [15]. Although such behaviours are essential for ensuring safe and coordinated care in high‐acuity settings [4], they may not directly enhance nurses' individual perceptions of job satisfaction. The demands of constant monitoring may add to cognitive workload or be perceived as routine professional responsibilities rather than inherently satisfying tasks. Although situation monitoring showed a significant positive association with job satisfaction in the bivariate analysis, this relationship was no longer significant when controlling for other relational and organisational variables in our multivariate model. This suggests that although situation monitoring may contribute to the broader teamwork process, it may not independently relate to ICU nurses' perceptions of job satisfaction when considered alongside factors more directly tied to interpersonal support and organisational safety. However, given its theoretical relevance in collaborative care environments, future studies should explore whether situation monitoring influences job satisfaction indirectly, or in interaction with contextual variables, such as workload and job strain.

## 7 Limitations

Examining multiple relational and organisational factors simultaneously, this study offers a broader perspective on job satisfaction among ICU nurses, an area that remains underexplored. However, several limitations should also be noted. First, the cross‐sectional design limits the ability to infer causal relationships; thus, the results should be interpreted as associations rather than causations. Second, data were collected through self‐reported questionnaires, which may be subject to response bias although anonymity and voluntary participation were ensured. Third, as the sample consisted exclusively of ICU nurses in Korea, the generalisability of the findings to other nursing populations or international healthcare settings may be limited. Finally, although the study examined several relational and organisational factors associated with job satisfaction, relationships with other roles in health care were not included beyond nurse–nurse and nurse–physician relationships. Given the complexity of ICU work environments and the diverse professional interactions within them, broader measures may better capture these nuanced dynamics. For example, the relational coordination scale assesses interdependent relationships among all roles involved in the specific care process of interest [38]. In the ICU, this might include physicians, nurses, respiratory therapists, pharmacists and case managers. In addition, other important variables, such as workload and career development opportunities [9, 11], were not included, which may have introduced unmeasured confounding.

## 8 Implications for Practice

The findings of this study provide practical implications for improving job satisfaction among ICU nurses. First, given the strong association between leader identification and job satisfaction, healthcare organisations should invest in leadership programmes for nurse managers. Programs that foster trust, mutual respect and alignment with staff values, such as leadership development workshops and mentorship programmes, can help strengthen leadership practices and competencies, which may ultimately improve leader identification [48, 49]. Second, the study reinforces the importance of a positive safety climate and reliable workplace safety systems. Healthcare organisations should establish clear safety protocols [50] and provide regular opportunities for staff to engage in discussions about workplace safety [51]. Third, to enhance collegial relationships between nurses and physicians, healthcare organisations should implement structured interventions, such as interdisciplinary simulation training and shared decision‐making, which actively involve both nurses and physicians in open discussions on patient care decisions based on each professional's expertise and perspectives [52, 53]. These efforts can help foster a sense of trust and support among nurses and other professionals, ultimately enhancing their job satisfaction.

## 9 Conclusion

This study underscores the significant relationships of relational and organisational factors with ICU nurses' job satisfaction. Specifically, strong identification with nurse leaders, collegial nurse–physician relationships, positive safety climate and well‐established safety systems were associated with higher levels of job satisfaction. These factors contribute to a more supportive and safe work environment in high‐stress ICU settings. To enhance nurses' job satisfaction, healthcare organisations should foster interprofessional collaboration, invest in leadership development and strengthen safety culture and systems. Addressing these areas may help improve workforce stability and the overall quality of patient care.

## Author Contributions

**Arum Lim:** conceptualisation, methodology, investigation, resources, writing – original draft, writing – review and editing. **Seung Eun Lee:** conceptualisation, resources, formal analysis, supervision, funding acquisition, writing – original draft, writing – review and editing.

## Funding

This work was supported by the National Research Foundation of Korea (NRF) grant funded by the Korean government (MSIT) (no.: RS‐2023‐00208138).

## Ethics Statement

The secondary data analysis was conducted in accordance with the principles of the Declaration of Helsinki and received ethics approval from the Yonsei University Health Systems Ethics Committee (no.: 4‐2024‐1406) on 31 December 2024.

## Consent

All participants were informed that their participation was voluntary and that they could withdraw from the study at any time without penalty. Informed consent was obtained electronically at the time of data collection.

## Conflicts of Interest

The authors declare no conflicts of interest.

## Data Availability

The data that support the findings of this study are available from the corresponding author upon reasonable request.
